# From ancestral knowledge to digital sales: analysis of the use of e-commerce in Amazonian indigenous communities in Peru

**DOI:** 10.12688/f1000research.176217.1

**Published:** 2026-02-16

**Authors:** VICTOR HUGO PUICAN RODRIGUEZ, José Luis Aguilar Delgado, María Cecilia Zavaleta López, Henry Elder Ventura Aguilar, Yoni Mateo Valiente Saldaña, Ena Cecilia Obando Peralta, Elizabeth Norma Calixto Arias, Tito Edinson Quispe Campos

**Affiliations:** 1 AMAZONAS, Fabiola Salazar Leguia National Intercultural University of Bagua, BAGUA, PERÚ, Peru; 2 LA LIBERTAD, National University of Trujillo, TRUJILLO, PERÚ, Peru; 3 LA LIBERTAD, National University of Trujillo, TRUJILLO, PERÚ, Peru; 4 LA LIBERTAD, National University of Trujillo, TRUJILLO, PERÚ, Peru; 5 LA LIBERTAD, National University of Trujillo, TRUJILLO, PERÚ, Peru; 6 LA LIBERTAD, Universidad Privada del Norte, Trujillo, La Libertad, Peru

**Keywords:** indigenous communities, Peruvian Amazon, available technology, e-commerce, technological interculturality

## Abstract

**Background:**

We explored the factors that influence the adoption of e-commerce and its link to digital purchasing decisions in indigenous Amazonian communities in Peru.

**Method:**

A quantitative approach was applied, with a non-experimental design and explanatory scope, based on 301 people aged 18 to 35, residents of Bagua. Surveys were used to evaluate dimensions such as performance expectations, effort, social influence, facilitating conditions, means of payment, available technology, and product variety.

**Results:**

The findings revealed low adoption of e-commerce associated with structural and sociocultural barriers, including lack of connectivity, limited technological knowledge, and limited training opportunities. Social influence and perceived usefulness were identified as key elements in promoting the use of digital platforms.

**Conclusion:**

A significant association was found between technological access, product variety, and the decision to purchase digitally, suggesting the need for contextualised strategies that respect cultural identity and enhance local capacities as a way to promote a sustainable and inclusive digital transition in indigenous communities in the Peruvian Amazon.

## I. Introduction

Over the last decade, digital transformation has redefined global business dynamics. In this process, e-commerce has gained prominence as an alternative and complementary sales channel, especially in contexts where physical restrictions, such as those caused by the pandemic, limited access to traditional establishments, driving the use of digital platforms in urban centres. However, it also posed new challenges and opportunities in rural and jungle areas of Peru, such as the city of Bagua, where indigenous producer communities and urban consumers coexist.

In this context, Berrío-Zapata et al. ^1^ reported that the health emergency has caused the temporary collapse of physical commerce, leading to a considerable increase in online shopping. However, there are still customers who experience barriers associated with mistrust, unfamiliarity, and the perception of virtual insecurity. For his part, Lacerda y Alves ^2^ mentioned that the lack of knowledge about the use of digital tools in e-commerce affected the growth of MSMEs, as they limited themselves to traditional sales because they did not have detailed knowledge of the benefits of e-commerce, both for offering products, promoting, building loyalty and online transactions.

According to Chu, ^3^ small artisans are at a disadvantage compared to large corporations due to various structural, financial, and technological limitations. At the same time, Annoni et al. ^4^ show that the rapid adoption of digital platforms requires an understanding of the motivations, needs, and expectations of digital consumers. Similarly, Ojeda et al. ^5^ pointed out that both technological advances and the internet brought about a process of creative destruction, as these changes gave rise to modern technologies that influence competitiveness, as well as benefits for businesses and e-commerce, thus transforming a chain of expansion, enabling all types of e-commerce transactions, as well as means of payment, becoming a viable channel for commerce.

Fan ^6^ meanwhile, identifies that the pandemic context was an opportunity for the growth of e-commerce; however, difficulties also arose related to consumer motivation to shop online, forcing firms and users to experiment with new platforms and digital purchasing processes in order to build trust and create satisfying experiences.

Theoretically, it is based on a theoretical approach that integrates models of technology adoption and digital consumer behaviour, as this allows for the analysis of the variables that influence the decision to purchase digitally. Methodologically, this study addressed a reality that has been little explored in the literature, given that it examines the use of e-commerce in rural Amazonian contexts where cultural tradition and emerging technologies converge. practically, the digital capabilities of local buyers were strengthened and the artisanal offerings of indigenous communities were made visible, promoting their integration into e-commerce as a means of economic development.

The objective was to explore the factors that influence the adoption of e-commerce and its influence on digital purchasing decisions in indigenous Amazonian communities in Peru. At the same time, the level of development of performance expectations, effort, social influence, facilitating conditions, means of payment, available technology, and product variety in association with the use of e-commerce in Amazonian communities was identified. At the same time, the association between the level of e-commerce adoption and digital purchasing decisions in indigenous communities was examined. in addition, the predictive influence of variables associated with e-commerce on digital purchasing decisions, means of payment, available technology, and perception of product variety was determined; finally, the barriers and opportunities faced by artisans in the transition to the use of e-commerce platforms were interpreted, taking into account their cultural context and level of technological familiarity.

Therefore, it was hypothesised that there is a significant relationship between the adoption of e-commerce and the variables associated with the decision to purchase digitally in Amazonian communities. Similarly, it was verified whether performance expectations, social influence and available technology have a significant influence on the decision to purchase digitally. In addition, it was examined whether enabling conditions have a significant negative influence on the adoption of e-commerce and the use of digital payment methods. Similarly, it was verified whether a greater perception of the usefulness of e-commerce increases the willingness to make online purchases. At the same time, it was evaluated whether greater technological availability leads to greater use of digital payment methods. Finally, it was shown whether the absence of facilitating conditions significantly reduces the likelihood of adopting digital commercial practices in Amazonian communities.

## II. Theoretical framework

E-commerce has transformed consumption patterns worldwide. In regions such as Bagua, which is part of the Peruvian jungle and characterised by its cultural diversity and the influence of indigenous communities, this change poses particular challenges given the impact of e-commerce on this local reality, where access to technology, connectivity and traditional customs influence the way people shop online. Ballerini et al. ^7^ revealed that virtual platforms improve consumer knowledge and allow firms to expand, especially from a managerial perspective. In the BRICS nations, Lei et al. ^8^ highlighted that, despite the growth of e-commerce, regional gaps and a lack of effective cooperation still persist. In the case of Bagua, where there are communities with traditional exchange practices, these gaps are particularly significant.

Michielsen et al. ^9^ developed a model that associates the use of platforms with repurchase and recommendation behaviour, which is useful for analysing how consumers in rural areas adapt to different types of e-commerce. For their part, Peña et al. ^10^ showed that purchase intent does not always translate into action, highlighting the influence of culture on impulse decisions, something that in Bagua may be linked to community norms or interpersonal trust. Kawasaki et al. ^11^ report that teleworking reduces the importance of the location of physical stores, a factor that in areas such as Bagua could reinforce the need for accessible digital channels. In the national context, Kumar et al. ^12^ found a strong association between e-commerce use and customer loyalty, suggesting opportunities for local businesses. Magro-Montero et al. ^13^ identified barriers to digital payments among people over 40, which could be replicated in communities with lower digital literacy.

From a theoretical perspective, the technology adoption model is considered, given that the behaviour used influences individual attitudes rather than behavioural intentions, which are influenced by expected benefit and perceived ease of use, which is divided into five constructs: performance expectations, effort expectations, social influence, facilitating conditions, and moderating aspects of influence (gender, age, and experience). ^14– 16^ According to Skare et al., ^17^ e-commerce is key to the growth of commercial systems, and the role of social and economic reforms in the intensified development of e-commerce and social networks has become increasingly prominent. On the other hand, He et al. ^18^ mentioned that the e-commerce platform is an emerging business model in which the e-commerce platform collects customer preference data and shares this information with manufacturers to facilitate product customisation. Furthermore, Degutis et al. ^19^ mentioned that e-commerce involves commercial transactions, which entails making commitments in a defined collaborative space between people using their IT systems. E-commerce logistics are e-commerce platforms that allow suppliers to set product prices in order to establish direct contact with consumers by paying a fee to the e-commerce platform, thus attracting potential customers through fast and efficient delivery, achieving accelerated growth in e-commerce platforms. ^20– 22^

According to Amezcua et al., ease of payment is important given the significant growth and technological advances in today's modern market, as banks work together with entities that supply goods and services to facilitate payments after purchases are made. This involves the use of systems and applications, this is why many banks make it easy for consumers to pay by mobile phone, online, using apps such as Plin, Yape and others, as well as paying on the companies' own platforms. This saves time and money and provides easy access regardless of where the buyer is located Amezcuna et al., ^23^ Melo & Jimenez ^24^ and Vollenwyder et al. ^25^ mentioned that web accessibility supports diversity and inclusion by preventing the emergence of barriers to web use that may result from sensory and cognitive impairments. Its goal is to provide useful information and services to as many people as possible and contributes to the role of the web in enabling and promoting equal opportunities in society. ^25, 26^

One of the theories taken into account when purchasing products online is the theory of planned behaviour, as it provides an interesting body of research for understanding why users shop online. This model is well developed in Information and Communication Technologies (ICT) research and can predict usage practices based on these planning theories. Here, human action is determined by the intention to perform that action. It is a function of attitudes and subjective norms and can be traced back to normative and behavioural ideologies. ^27– 30^

On the other hand, Das et al. ^31^ mentioned that in online shopping there is a perception of purchasing methods, the main factor being trust, since it does not depend on direct customer service from the seller, but rather on the perception of the advantages, convenience and security of the place of purchase. Likewise, Salas et al. ^32^ indicated that payment methods tend to be electronic and are divided into significant payments and minimal payments, both of which are equally important in influencing the demand for money. In addition, it can explain the mechanisms that monetary authorities can use as regulators to maintain the efficiency and stability of electronic payment methods, thus giving confidence to more economic actors to carry out any electronic transaction. Bermeo et al. ^33^ also mentioned that online means of payment are transactions that accept electronic money and are carried out using online means of payment that highlight mistrust, negative attitudes, management, promotions and security. Finally, Shakeel & Khan ^34^ stated that payment methods will improve both in terms of security and information privacy, which are the weaknesses of using virtual means to make payments. They proposed and tested a security protocol as a technical solution for mobile payment transactions using virtual means to make payments.

According to Wan et al., ^35^ new technologies such as virtual reality, artificial intelligence (AI), machine learning, blockchain, cloud computing, the Internet of Things (IoT) and other technological advances often arise from the desire to gain a competitive advantage. Furthermore, Galvez et al. ^36^ indicated that technology has significant value for organisations, but the scope and dimensions of its impact are strongly influenced by internal and external factors, such as the complementary resources of the organisation and its partners, and the competitive environment of the company. Finally, Brey ^37^ mentioned that technology provides fundamental support for well-being and justice as intrinsic values for a good society, and also defends the necessary instrumental values, including freedom, democracy, and sustainability.

According to Lyons et al. ^38^ product variety is the expansion of products offered in the market to give customers more options in the same establishment, satisfying their needs. Likewise, Wan et al. ^35^ pointed out that in a market where there is high demand, product variety is essential for growth, and increases in product variety lead to higher inventory levels because more inventory is needed to compensate for the loss of demand. Finally, Dominik & Groll ^39^ state that product variety is a growing trend of offering highly configurable products, but it increases complexity costs throughout the product life cycle. According Carrión, ^40^ stock or inventory is the existence of goods, and inventory management is essential for trade. It also refers to the products, goods, or services that are available and stored in a specific location to be sold during the course of the day. In addition, Carrión ^40^ mentioned that product turnover is how many times a product has been sold in a given period of time and the number of times it moves in and out of inventory. Finally, Chang & Wang ^41^ pointed out that diversification is any company that creates new consumer products or new services on different platforms, whether they are existing products that have been improved and targeted at a specific group of consumers.

## III. Methodology

The link between e-commerce and online shopping was understood and explored in depth in order to describe possible solutions to the phenomena detected, as detailed by CONCYTEC. ^42^ It was also based on the collection and processing of numerical data, allowing for the objective measurement and analysis of consumer behaviour patterns through the information obtained, as reported by Neira et al. ^43^; at the same time, the variables were not deliberately manipulated, but rather observed as they occur in their natural context; Furthermore, the data were collected at a single point in time, as this facilitated a precise description of the situation observed. Simultaneously, the main characteristics of users in Bagua were described, and the causal association between the study variables was found, as revealed by. ^44, 45^

The universe consisted of 1,370 people between the ages of 18 and 35, residing in Bagua, selecting those who have greater access to technology and are in frequent contact with digital platforms. At the same time, consideration was given to those who were between the ages of 18 and 35, also residing in Bagua, and above all, those who agreed to participate voluntarily in the study. On the other hand, individuals who did not meet the age range or who decided not to respond to the questionnaire, either in person or online, were excluded.

To determine the sample, the finite proportion formula was applied, resulting in 301 people between the ages of 18 and 35 who reside in Bagua. The data collection technique was a survey, due to its usefulness in obtaining direct data from participants in a short time. At the same time, a 40-item questionnaire was used as the instrument, divided into twenty items for each variable. It was prepared in digital format (Google Forms). Similarly, the validity of the instrument was verified by four experts in the field, who reviewed the consistency, clarity, and relevance of each question. A pilot test was also conducted to verify the reliability of the questionnaire and make the necessary adjustments before its final application.

The ethical principles required by UNIFSL in all its research will be taken into account; therefore, autonomy is considered because the people who will participate in answering the questionnaire will have the ability to choose whether or not to participate; in addition, beneficence is taken into account because this research seeks the well-being of each participant. Likewise, human integrity is taken into account, since this work recognises and prioritises the interests of individuals over those of science. Similarly, justice is taken into account, since all those who participate in this research will be treated equally without any discrimination.

**Figure 1.**
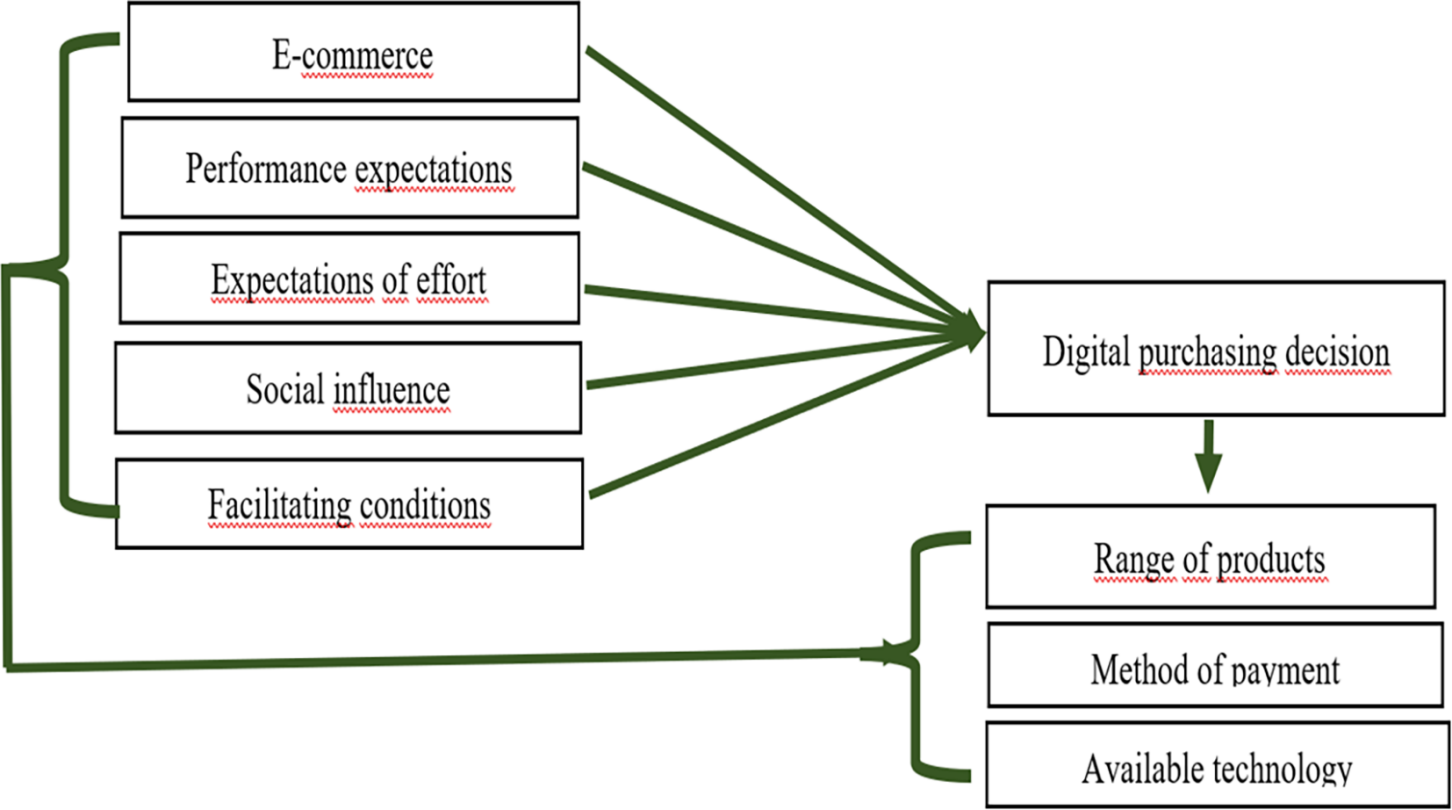
Theoretical model of the variables that influence digital purchasing decisions in indigenous Amazonian communities in Peru. **Source:** Own elaboration based on data from the literature review.

Figure 1 shows the proposed theoretical model, which was constructed using an explanatory approach that groups individual and contextual variables associated with digital purchasing behaviour in indigenous communities in the Peruvian Amazon. This model is based on the main variable of e-commerce, which acts as a cross-cutting axis and directly influences digital purchasing decisions. At the same time, performance expectations, effort expectations, social influence, and facilitating conditions were incorporated as predictor variables, all of which are fundamental to the technology acceptance model (UTAUT) and adapted to the intercultural context. The digital purchasing decision is connected to specific dependent variables such as product variety, payment methods, and available technology, which provide feedback to the process through a relational flow. This methodological design made it possible to identify both barriers and opportunities for digital transformation in areas with limited access to technology and was validated through logistic regression analysis and non-parametric correlation, ensuring the relevance of the model in contexts with high sociocultural diversity.

This study involved human subjects and was conducted in accordance with the ethical principles of the Declaration of Helsinki, whereby participants were informed about the confidentiality of their data and the voluntary nature of their participation, and were asked to sign a consent form. The study was conducted using an anonymous in-person and online questionnaire, and the risk to subjects was considered minimal. The authors' institution does not currently have a formal Research Ethics Committee/IRB that grants approvals or exemptions for this type of research, so no approval, exemption, or reference number can be provided. However, standard ethical safeguards were applied throughout the research: clear information was provided about the purpose and procedure of the study; participation was entirely voluntary; and the right to refuse or withdraw from participation at any time, without penalty or sanction, was guaranteed. No personal information was collected. Responses were treated anonymously and confidentially, stored securely with access restricted to the research team, and used solely for academic purposes. Results are presented in aggregate form only to prevent identification of participants.

All subjects were informed about the study and gave written informed consent before participating. Written consent was obtained and contained information on study purpose, intensity of participation, and the voluntary shield right to withdraw at any time without having to state reasons. All participants were ≥18 years of age. No incentives were offered, and no personally sensitive identifiers were collected.

## IV. Results

**Figure 2.**
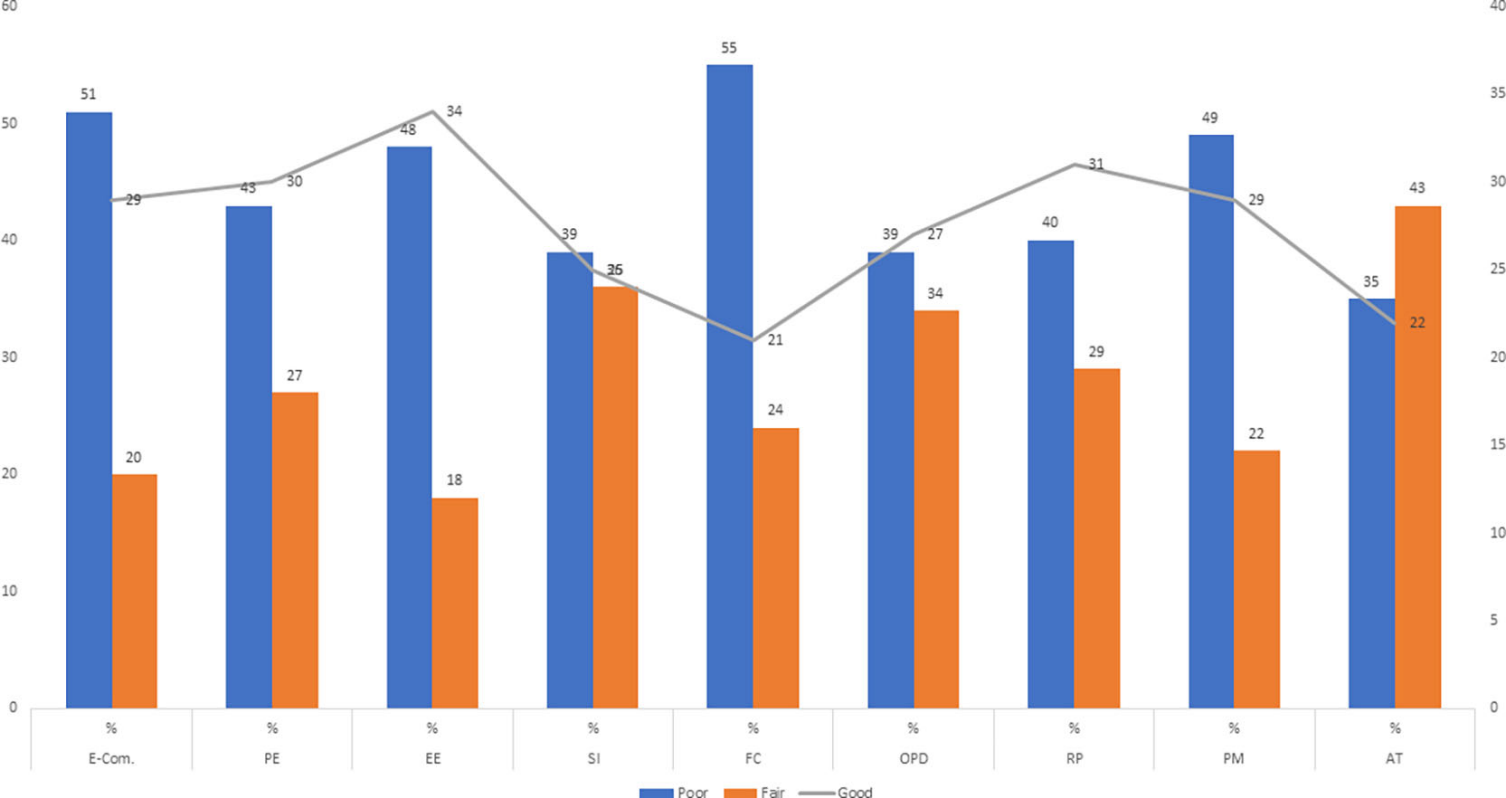
Percentage distribution of response levels in the variables of the study on e-commerce and digital purchasing decisions in Amazonian communities in Peru. Note: E-Com.: e-commerce; ED: Performance expectations; EE: Effort expectations; IS: Social influence; CF: Facilitating conditions; OPD: Online purchasing decision; VP: Product variety; PM: Payment method; TD: Available technology.

In Figure 2, the data from the results confirm that more than half of this sample has a deficient level of e-commerce adoption, with an alarming 51%. At the same time, it shows high scores in enabling conditions, with 55%, in effort expectations, with 48%, and in technology, with 49%. This suggests the presence of worrying barriers to the implementation of e-commerce, such as inadequate digital infrastructure, lack of training in digital skills and unfavourable institutional support. In terms of payment methods, 40% were found to be above average, as were online purchasing decisions, at 39%. It can be inferred that the lack of skills to execute secure and reliable online transactions conditions the participation of these actors in virtual commerce.

Table 1 shows that the Kolmogorov-Smirnov normality test revealed that the variables show significance values below 0.05, which indicates that they do not follow a normal distribution, justifying the use of non-parametric statistics for inferential analysis. At the same time, a high deviation from normality is observed, which highlights that there is heterogeneity in perceptions of e-commerce in Amazonian indigenous communities, pointing to a complex structure of barriers and opportunities that behaves in a homogeneous manner; this behaviour is relevant as it reflects a diverse and challenging scenario that requires differentiated and culturally contextualised strategies to promote digital economic development in these communities.

**Table 1.** Kolmogorov-Smirnov normality test for the study variables.

	Kolmogorov-Smirnov ^a^		
	Statistician	gl	Sig.
E-Com.	0.325	301	0.000
PE	0.279	301	0.000
EE	0.316	301	0.000
SI	0.251	301	0.000
FC	0.345	301	0.000
PM	0.264	301	0.000
AT	0.313	301	0.000
RP	0.230	301	0.000
OPD	0.254	301	0.000

Note: E-Com: E-Commerce; PE: Performance expectations; EE: Effort expectations; SI: Social influence; FC: Facilitating conditions; OPD: Online purchasing decision; PM: Payment methods; AT.: Available technology; PV: Product variety.

The Table 2 reveals positive and significant correlations between e-commerce (E-Com) and all the variables analysed; the strongest associations are with performance expectations (ED: p=0.824), effort expectations (EE: p=0.694) and social influence (IS: p=0.647), suggesting that perceived ease, social support and trust are essential for the adoption of e-commerce in indigenous communities; the link with enabling conditions (EC: p=0.624) and technology (Tech: p=0.624) is also high, revealing structural barriers that still exist; the decision to purchase online (DCI) is strongly related to product variety (VP: p=0.842) and technological access (Tec.: p=0.771), which points to key opportunities to strengthen the digital offering and infrastructure in these territories.

**Table 2.** Spearman's correlation matrix between e-commerce and associated variables.

			E-Com	PE	EE	SI	FC	PM	AT	RP	DPD
Rho de Spearman	E-Com.	1									
	ED	0.824	1								
	EE	0.694	0.840	1							
	IS	0.647	0.770	0.794	1						
	CF	0.624	0.735	0.776	0.776	1					
	MP	0.627	0.721	0.663	0.663	0.607	1				
	TD.	0.624	0.647	0.582	0.582	0.490	0.605	1			
	VP	0.386	0.432	0.406	0.406	0.308	0.502	0.595	1		
	DCI	0.543	0.587	0.554	0.554	0.451	0.605	0.771	0.842	1	

Note: E-Com: E-Commerce; PE: Performance expectations; EE: Effort expectations; SI: Social influence; FC: Facilitating conditions; OPD: Online purchasing decision; PM: Payment methods; AT.: Available technology; PV: Product variety.

The data in Table 3 reveal that enabling conditions have a clear and significant negative effect on all dependent variables; that is, when the environment does not offer adequate support, such as internet access, technology, or training, the probability of using payment methods, technology, shopping online, or finding a variety of products decreases. At the same time, social influence has a positive effect, especially on the use of technology and purchasing decisions, because recommendations from close friends or local leaders increase the adoption of digital tools. Similarly, performance expectations also have an impact, but a negative one, since when users do not believe that e-commerce will be beneficial, the probability of using it decreases, especially in terms of payment methods and technology. likewise, the group with efficient use of e-commerce=1 is more likely to shop online and use technology; although not all relationships are significant, the findings show that the greater the familiarity with digital technology, the greater the participation in these practices; Finally, in the second model (category 2 versus 3), only social influence on payment methods and, to a lesser extent, on product variety, shows clear effects, revealing that intermediate perceptions have a less defined impact.

**Table 3.** Results of the logistic regression model for variables associated with digital purchasing decisions, payment methods, technology, and product variety.

	Detail	DPD			PM			RP			AT		
		B	Sig.	Exp(B)	B	Sig.	Exp(B)	B	Sig.	Exp(B)	B	Sig.	Exp(B)
1	Intersection	3.331	0.012		6.233	0.000		3.586	0.004		4.048	0.004	
	PE	-1.053	0.066	0.349	-1.812	0.002	0.163	-1.575	0.004	0.207	-1.098	0.063	0.334
	EE	-0.255	0.558	0.775	-0.199	0.656	0.820	-0.267	0.523	0.766	-0.054	0.904	0.948
	SI	0.934	0.041	2.545	0.090	0.853	1.094	1.034	0.022	2.812	0.753	0.098	2.124
	FC	-2.118	0.000	0.120	-1.900	0.000	0.150	-1.313	0.001	0.269	-1.712	0.000	0.181
	E-Com.= 1]	1.750	0.042	5.754	0.679	0.437	1.972	1.273	0.095	3.570	0.598	0.474	1.818
	E-Com.=2]	1.111	0.149	3.038	-0.091	0.914	0.913	-0.120	0.863	0.887	0.151	0.826	1.163
	E-Com.=3]	0 ^b^			0 ^b^			0 ^b^			0 ^b^		
2	Intersection	2.040	0.092		0.546	0.656		1.731	0.164		3.632	0.006	
	PE	-0.495	0.335	0.610	-0.367	0.452	0.693	-0.692	0.182	0.501	-0.340	0.535	0.712
	EE	-0.035	0.926	0.965	-0.589	0.113	0.555	0.015	0.969	1.015	0.100	0.809	1.105
	SI	-0.219	0.563	0.804	0.799	0.041	2.223	0.143	0.715	1.154	-0.910	0.026	0.403
	FC	-0.236	0.474	0.790	-0.356	0.248	0.701	-0.364	0.269	0.695	-0.352	0.323	0.703
	E-Com.= 1]	0.858	0.232	2.357	1.230	0.068	3.421	-0.130	0.861	0.878	0.613	0.425	1.845
	E-Com.=2]	0.282	0.598	1.326	0.599	0.253	1.820	0.182	0.741	1.199	-0.090	0.875	0.914
	E-Com.=3]	0 ^b^			0 ^b^			0 ^b^			0 ^b^		

Note: E-Com: E-Commerce; PE: Performance expectations; EE: Effort expectations; SI: Social influence; FC: Facilitating conditions; OPD: Online purchasing decision; PM: Payment methods; AT.: Available technology; PV: Product variety.

## V. Discussion

The results show that e-commerce is not yet common practice in the Amazonian communities evaluated, given that more than half of the participants reported a low level of adoption, pointing to persistent limitations in access, training, and digital trust. this situation is exacerbated by poor infrastructure and limited institutional support, reducing the ability to take advantage of the benefits offered by e-commerce in territories with their own cultural identity; at the same time, expectations of performance and effort, together with enabling conditions, scored high percentages at deficient levels, showing that many people feel that shopping online is complicated or of little use; Similarly, the lack of networks, connectivity and technological tools prevents active participation. Supported by research by Ballerini et al., ^7^ and Peña et al., ^10^ the perception of difficulty discourages the intention to use e-commerce, especially in rural contexts with less digital exposure.

In another sense, the correlations reveal that when clear advantages are perceived and social support is available, interest in e-commerce increases, given that performance expectations were positively and strongly related to the use of e-commerce, which shows that perceived usefulness is a key factor. Similarly, social influence was significant, which coincides with the findings of Lei et al., ^8^ highlighting the value of recommendations in purchasing decisions, especially in communities where relationships of trust are central.

A significant connection was also found between the decision to shop online and product variety, suggesting that when people find more options, they feel more motivated to shop online. At the same time, the availability of technology improves this decision, as demonstrated by findings that coincide with those of Wang et al., ^45^ Kawasaki et al. ^11^ and Michielsen et al., ^9^ who highlighted the importance of a wide and accessible range of products to stimulate online consumption.

In the regression model, it was found that facilitating conditions have a negative influence on all the variables analysed; this means that when the environment does not provide support such as internet access or training, the probability of using digital platforms decreases, as supported by Magro-Montero et al. ^13^ on structural barriers in remote areas. On the other hand, social influence is once again shown to be positive, especially in the use of technology and in purchasing decisions, highlighting the role of close networks in the adoption of new practices, which is encouraging. For its part, the group that showed greater familiarity with e-commerce also showed a greater willingness to use digital payment methods and purchase products online. Although not all relationships were statistically significant, a trend was confirmed: the greater the experience, the greater the participation. The data is supported by Skare et al., ^17^ who emphasise that the repetition of successful digital experiences strengthens user confidence.

Finally, the intermediate categories showed fewer clear relationships. This could indicate that there is a segment of the population with undecided positions or in the process of adaptation; for this group, interventions must be more personalised, practical, and culturally relevant, suggesting that the implementation of e-commerce in Amazonian communities requires more than infrastructure, needing human support, respect for local culture, and strategies designed based on the reality of the territory.

## VI. Conclusions

The data obtained allows us to conclude that e-commerce in the Amazonian communities studied is in a limited stage of development, marked by restrictions that go beyond access to the internet or devices, because there is a combination of social, cultural and structural factors that prevent these digital tools from being smoothly integrated into local productive life. Although people are familiar with the technology, but do not always understand it as an ally for improving their economic activities, and this is reflected in low levels of adoption and perceptions of difficulty or mistrust.

The absence of enabling conditions, such as infrastructure, technical guidance or contextualised training, has a direct impact on low participation in digital sales spaces, because without support, without a support network and without the right tools, it is understandable that e-commerce is not perceived as a real possibility, since it is not just a matter of installing the internet or providing equipment; it is about creating environments of trust, where people can learn without fear and experiment without feeling overwhelmed, given that technology without cultural meaning tends not to take root.

Despite this outlook, the study shows clear signs of opportunity, given that people who recognise the practical value of e-commerce or who receive recommendations from those close to them show a greater willingness to use it. At this point, social influence and expectations of benefit become drivers of change, since if the community supports the process and users perceive that there is a real gain, interest grows. At the same time, it was evident that when there are more product options and a certain familiarity with the use of technology, the intention to purchase digitally improves.

The analysis also suggests that not everyone is at the same stage of adoption, as there are segments within the community with greater willingness or experience that could play a key role in strengthening collective capacities. these profiles become local references if they are offered adequate support; similarly, it is confirmed that a strategy focused solely on infrastructure is not enough; therefore, an approach that considers the rhythms, values and logic of the territory is recommended, because it is not enough to teach how to use a platform, it is necessary to link it to the identity and history of what is produced.

The study provides a perspective from the territory, where e-commerce is not presented as a universal formula, but rather as a tool that must be adapted to the social and cultural environment; where its successful incorporation will depend on the ability to connect local knowledge with digital dynamics; Therefore, it is suggested that practical training processes be promoted, designed based on knowledge of the context, with ongoing support and accessible formats, where partnerships with universities, municipalities, or technological entities can facilitate this process if they are geared towards strengthening local capacities.

It is also important to promote digital platforms that not only sell products, but also communicate their history, symbols and roots, because the variety of the offer, together with the narrative of identity, turns each product into a bridge between the ancestral and the contemporary. Similarly, it is necessary to continue researching, through dialogue between disciplines, how to bring digital development to these territories without erasing their essence, but rather strengthening it, since the real challenge is not only to connect to the network, but to connect to the future without losing sight of who we are.

## Ethics and consent

Ethical approval for the article ‘From ancestral knowledge to digital sales: analysis of the use of e-commerce in Amazonian indigenous communities in Peru’ was granted by the Ethics Committee of the Fabiola Salazar Leguía National Intercultural University of Bagua. The committee verified the protocol, instruments, informed consent procedures, and authorisation to conduct the study in its Requirements Verification Report dated 11 May 2024.

The university does not assign an independent IRB/ethics code beyond the committee's verification and the university's resolution; therefore, no additional approval number is available. Written informed consent was obtained from all participants prior to data collection; all respondents were ≥18 years of age. The study complied with the Declaration of Helsinki and applicable national and institutional research ethics standards. No independent external review committee was involved; ethical oversight was provided internally by the university committee.

## Additional materials and data

As part of our commitment to methodological transparency and scientific rigour, the study is supported by a set of additional materials and data that complement the information presented in the main manuscript. Firstly, there is a file containing the complete database used in the analysis, in which the variables and dimensions considered in the research are systematised, allowing for a more detailed understanding of the process of treatment and analysis of the information collected.

It also includes a file containing supplementary statistical tables, which provide a more detailed breakdown of the descriptive and inferential results obtained. These tables provide a greater level of analytical detail and facilitate the verification of the findings presented, without overloading the main body of the article.

In addition, two graphic files are included that visually represent the main results of the study. These figures have been prepared from the analysed data and serve an interpretative function, synthesising and reinforcing the most relevant patterns and relationships identified in the statistical analysis.

Together, these materials are available in the same data repository and provide essential support for a comprehensive understanding of the study, promoting replicability, critical review, and responsible use of the information for academic and scientific purposes.

**Descriptive results figures**

Puican Rodríguez, VH, Aguilar Delgado, JL, Zavaleta López, MC, Ventura Aguilar, HE, Valiente Saldaña, YM, Obando Peralta, EC, Calixto Arias, EN, and Quispe Campos, TE (2025). Descriptive results figures. B2SHARE. https://doi.org/10.23728/b2share.wdpv2-7cm53 ^46^

**Data base y tables**

**Version 1**

Puican Rodriguez, V. H., Aguilar Delgado, J. L., Zavaleta López, M. C., Ventura Aguilar, H. E., Valiente Saldaña, Y. M., Obando Peralta, E. C., Calixto Arias, E. N., & Quispe Campos, T. E. (2026). DATA BASE, TABLES AND QUESTIONNAIRE. B2SHARE. https://doi.org/10.23728/b2share.vxbwt-gnt24 ^48^

**Process identifier**

The materials associated with this study are duly identified and organised in an open-access data repository, allowing for transparent review and independent replication of the research process. The complete dataset is available without restrictions or embargo periods, under a Creative Commons Attribution 4.0 (CC-BY 4.0) licence, guaranteeing its free consultation, use and reuse for academic and scientific purposes, provided that the corresponding authorship is acknowledged.

## Data Availability

The underlying data supporting the results of this study are publicly available at: **Descriptive results figures** DOI: https://doi.org/10.23728/b2share.wdpv2-7cm53 ^46^ **Data Base, Tables and Questionnaire** **Version 1** DOI: https://doi.org/10.23728/b2share.c0x51-tg219 ^47^ **Version 2** DOI https://doi.org/10.23728/b2share.vxbwt-gnt24 ^48^ Under license [ Creative Commons Attribution 4.0 International/ Creative Commons Zero v1.0 Universal]. The repository includes the dataset necessary to replicate all the results presented, including: (i) the values underlying the descriptive statistics (e.g., means, standard deviations, and related measures); (ii) the values used to generate figures and tables; (iii) the complete set of observations ready for analysis; and (iv) a data dictionary/code book describing all variables and dimensions (the responses to each item by the respondents involved). The dataset is available without restrictions and does not require login access.
