# Supplementary material for: From ancestral knowledge to digital sales: analysis of the use of e-commerce in Amazonian indigenous communities in Peru
Source: F1000Res. 2026 Feb 16;15:278. [Version 1] doi: 10.12688/f1000research.176217.1 (PMC13182782; doi:10.12688/f1000research.176217.1)
Supplement: Peer review history

# Reviewer report: Reviewer response for version 1

**DOI:** 10.5256/f1000research.194254.r462389

**Published:** 2026-03-28

**Authors:** Laxmiram Gope, SHAKTIPADA MAHATO

**Affiliations:** 1 Assistant Professor, Department of Education., Sidho-Kanho-Birsha University, Lagda, West Bengal, India; 2 Research Scholar, Department of Education,, Sidho-Kanho-Birsha University, Purulia, West Bengal, India

At the outset, I express my heartfelt thanks for the wonderful opportunity to review this article.

 First, the study's title is well-crafted and attractive.

 Suggestion for improvement:

 a. Before the method of the study within the abstract, the objective of the study should be clearly stated and mentioned.

 b. Although the method section is well crafted and point-specific. Hence, the methodological table may be incorporated at the end of the study.

 c. The study's findings are well reported, specific, and well represented, but they could be presented in tabular form aligned with the work's objectives.

 d. But ultimately, the study's findings address the research gap and contribute to a new knowledge paradigm.

 e. Implications of the study: As an indigenous knowledge worker, I agree that this study meets the academic integrity of the knowledge paradigm. At this juncture, preservation, promotion, and passing down(3'p') of indigenous knowledge became the fundamental challenges. Hence, through the digitalization of these indigenous knowledge practices, we were able to easily utilize them. Through digitalization, such a knowledge paradigm has many implications for modern IPR(Intellectual Property Rights) among the indigenous communities across the globe and it will also become a new dimension of knowledge cultivation and applications. From the ancestral knowledge perspective, it can be said that the indigenous cum ancestral knowledge has many applications for sustainability in the context of social, economic, and environmental considerations.

 Therefore, from the above perspective, it can be concluded that this research study has many implications for indigenous communities and for environmental and economic sustainability.

Is the work clearly and accurately presented and does it cite the current literature?

Yes

If applicable, is the statistical analysis and its interpretation appropriate?

Yes

Are all the source data underlying the results available to ensure full reproducibility?

Yes

Is the study design appropriate and is the work technically sound?

Yes

Are the conclusions drawn adequately supported by the results?

Yes

Are sufficient details of methods and analysis provided to allow replication by others?

Partly

Reviewer Expertise:

Indigenous Knowledge,  Sustainability Education, Development

We confirm that we have read this submission and believe that we have an appropriate level of expertise to confirm that it is of an acceptable scientific standard.

# Reviewer report: Reviewer response for version 1

**DOI:** 10.5256/f1000research.194254.r460134

**Published:** 2026-03-06

**Authors:** Glen Maimela

**Affiliations:** 1 Research and Knowledge Production, National Heritage Council (Ringgold ID: 450776), Pretoria, Gauteng, South Africa

**Introduction**

 In the second paragraph, second sentence, the authors introduce the abbreviation MSMEs. Since it is the first time being mentioned in the study, I suggest the authors write it in full first and then later use the abbreviation.

**Theoretical framework**

 The authors critically outlined the theoretical frameworks that underpinned the study. Although the theoretical framework is well outlined. I suggest the following

- In the first paragraph under the theoretical framework, the acronym BRICS can be written in full first.
- In the second paragraph under the theoretical framework, the authors mentioned “Michielsen et al. developed a model that associates the use of platforms with repurchase and recommendation behaviour, which is useful for analysing how consumers in rural areas adapt to different types of e-commerce”. From the readers’ point of view, this sentence keeps the readers wondering about the model developed. What is the name of it and what does it say?
- In the fourth paragraph under the theoretical framework, the numeric referencing for “Amezcua et al.” is missing.
- The technology adoption model and the theory of planned behavior can be identified from the section as model/theories used for the study. Some of the readers may know a little about these theories, I therefore, suggest the authors consider giving a succinct background of these theories. This can be – Who developed the theory and when, what does the theory say? These key will be crucial in linking the theories with the study. Before linking the theories to the study, the authors should consider this example:  “The Diffusion of innovations theory was considered for this study. The theory is a brainchild of Everett Rogers in 1962, which seeks to understand how people adopt new technological developments in their lives.”

**Methodology**

- In the first paragraph under methodology, “CONCYTEC” can be written in full first before being used as an abbreviation. The same can be done for “UNIFSL” in the fourth. paragraph.
- The fourth paragraph is written in the future tense, highlighting that the study data is not yet collected or analysed. I therefore suggest the authors write this section using the past tense.

**Discussion**

- The discussion is informative, linking the study findings with previous studies. However, the discussion can be improved by linking some of the key findings with the theoretical perspectives outlined in the theoretical framework (Technology Adoption Model and the Theory of Planned Behavior)

**Conclusion**

 The conclusion outlines practical training, promotion of digital platforms and further research as suggestions that can improve access and use of e-commerce. In addition to future research, the study can suggest long-term impact studies that will assess the impact of e-commerce. Additionally, a recommendation along policy development and refinement can be beneficial for the government level to consider for increasing access to e-commerce in the Amazonian Indigenous communities.

Is the work clearly and accurately presented and does it cite the current literature?

Yes

If applicable, is the statistical analysis and its interpretation appropriate?

Yes

Are all the source data underlying the results available to ensure full reproducibility?

Yes

Is the study design appropriate and is the work technically sound?

Yes

Are the conclusions drawn adequately supported by the results?

Yes

Are sufficient details of methods and analysis provided to allow replication by others?

Yes

Reviewer Expertise:

Digitisation, Indigenous knowledge,

I confirm that I have read this submission and believe that I have an appropriate level of expertise to confirm that it is of an acceptable scientific standard.
